# Simple Quantitative Sensory Testing Reveals Paradoxical Co-existence of Hypoesthesia and Hyperalgesia in Diabetes

**DOI:** 10.3389/fpain.2021.701172

**Published:** 2021-06-28

**Authors:** Line Elise Møller Hansen, Camilla Ann Fjelsted, Søren Schou Olesen, Anna Evans Phillips, Mahya Faghih, Anne-Marie Wegeberg, Asbjørn Mohr Drewes, Christina Brock

**Affiliations:** ^1^Mech-Sense, Department of Gastroenterology and Hepatology, Aalborg University Hospital, Aalborg, Denmark; ^2^Centre for Clinical Research, North Denmark Regional Hospital, Hjoerring, Denmark; ^3^Department of Clinical Medicine, Clinical Institute, Aalborg University, Aalborg, Denmark; ^4^Centre of Pancreatic Diseases, Aalborg University Hospital, Aalborg, Denmark; ^5^Division of Gastroenterology, Hepatology, and Nutrition, Department of Medicine, University of Pittsburgh School of Medicine, Pittsburgh, PA, United States; ^6^Division of Gastroenterology and Hepatology, Department of Medicine, Johns Hopkins Medical Institutions, Baltimore, MD, United States; ^7^Steno Diabetes Center Nordjylland, Aalborg, Denmark

**Keywords:** diabetes mellitus, diabetic neuropathy, quantitative sensory testing, hypoesthesia, hyperalgesia

## Abstract

**Background:** Diabetic neuropathy is characterized by the paradoxical co-existence of hypo- and hyperalgesia to sensory stimuli. The literature shows consistently sensory differences between healthy and participants with diabetes. We hypothesized that due to differences in pathophysiology, advanced quantitative sensory testing (QST) might reveal sensory discrepancies between type 1 (T1D) and type 2 diabetes (T2D). Furthermore, we investigated whether vibration detection thresholds (VDT) were associated with sensory response.

**Method:** Fifty-six adults with T1D [43 years (28–58)], 99 adults with T2D [65 years (57–71)], and 122 healthy individuals [51 years (34–64)] were included. VDT, pressure pain detection thresholds (pPDT) and tolerance (pPTT), tonic cold pain (hand-immersion in iced water), and central pain mechanisms (temporal summation and conditioned pain modulation) were tested and compared between T1D and T2D. VDT was categorized into normal (< 18 V), intermediary (18–25 V), or high (> 25 V).

**Results:** In comparison to healthy, analysis adjusted for age, BMI, and gender revealed hypoalgesia to tibial (pPDT): *p* = 0.01, hyperalgesia to tonic cold pain: *p* < 0.01, and diminished temporal summation (arm: *p* < 0.01; abdomen: *p* < 0.01). In comparison to participants with T2D, participants with T1D were hypoalgesic to tibial pPDT: *p* < 0.01 and pPTT: *p* < 0.01, and lower VDT: *p* = 0.02. VDT was not associated with QST responses.

**Conclusion:** Participants with T1D were more hypoalgesic to bone pPDT and pPTT independent of lower VDT, indicating neuronal health toward normalization. Improved understanding of differentiated sensory profiles in T1D and T2D may identify improved clinical endpoints in future trials.

## Introduction

Diabetes mellitus comprises a number of metabolic diseases, including type 1 diabetes (T1D) characterized by autoimmune destruction of pancreatic β-cells and insulin deficiency, and type 2 diabetes (T2D) characterized by varying degrees of insulin resistance, insulin deficiency, and low-grade inflammation [1, 2]. The global prevalence of diabetes increases along with accompanying macro and microvascular complications affecting multiple organ systems [3–5]. One of the most common microvascular complications is distal symmetrical polyneuropathy (DSPN), which affects up to 50% of adults with long-term diabetes and leads to structural and functional damage of peripheral nerves and neurons of the central nervous system [6, 7]. Symptoms paradoxically range from numbness or hypoalgesia (non-painful DSPN) to allodynia or hyperalgesia (painful DSPN), with ~1/3rd developing neuropathic pain in the presence of DSPN [8, 9]. In recent years, DSPN-focused research has evolved from a glucocentric viewpoint to a broader understanding of the underlying pathophysiology secondary to multiple linked metabolic and inflammatory insults. Evidence that low-grade inflammation plays an important role in the pathogenesis of DSPN is emerging from both experimental and clinical studies [6, 10]. Consequently, differences in sensory responses may exist between T1D and T2D, plausibly due to varying degrees of neuro-inflammation.

Apart from nerve conduction studies, which are the gold standard to diagnose DSPN, uncomplicated clinical testing methods are needed to characterize DSPN [1]. A commonly used but simple bedside tool is vibration detection threshold (VDT), which primarily investigates the function of peripheral Aβ-nerves and thus is considered a clinical proxy for large fiber function [11]. Quantitative sensory testing (QST) is a commonly used research tool that may elucidate the involvement of specific pain mechanisms and underlying nerve functions in health and, e.g., diabetic neuropathy. QST has previously been used to investigate and demonstrate different sensory phenotypes (painful vs. non-painful diabetic neuropathy) aiming for targeted stratified analgesic treatment [12, 13]. The dynamic QST sensory profile in non-painful diabetic neuropathy has primarily been characterized in mixed T1D and T2D cohorts [14–18]. Furthermore, it is unclear whether neuronal alterations in non-painful diabetic neuropathy are caused by peripheral sensitization, transmission, through alterations of central processing, or a combination of these mechanisms.

We originally developed a specialized QST-protocol for chronic pancreatitis, which reliably characterizes peripheral and central pain mechanisms [19, 20]. The protocol includes validated specific phasic pressure tests, repetitive tests to assess temporal summation (TS), tonic cold pain (summated pain response to the immersion of the hand into cold water), and capacity of conditioned pain modulation (CPM). TS and CPM investigate primarily central pain mechanisms [21]. TS is defined as increased pain caused by increased C-fiber input to repetitive nociceptive stimulations and reflects central neuronal excitability. CPM is based on the phenomenon “pain inhibits pain” [22] and is a proxy of descending inhibition, i.e., how brainstem centers can gate the afferent neuronal barrage at the spinal cord level. This phenomenon leads to exaggerated pain experiences [23]. Sparse data indicate that TS is affected in diabetes, while CPM has shown to be impaired in patients with painful DSPN in comparison to non-painful DSPN or healthy participants [9, 24–26]. However, it is unknown whether the sensory profile is altered before the onset of clinical signs and symptoms of neuropathy. It has been described that a systematic shift seems to appear manifesting to hyperalgesia for nociceptive and hypoesthesia for non-nociceptive parameters [16]. Thus, an improved understanding of the somatosensory abnormalities within different types of diabetes may be important in order to identify future primary endpoints in clinical trials.

Based on the difference in neuro-inflammation, we hypothesized that the sensory profile would differ between participants with T1D and T2D, in terms of altered responses to QST, and that VDT was associated with the sensory response. Hence, the aims were: (1) to characterize the response to peripheral phasic and tonic pain stimuli; (2) investigate central pain mechanisms exploring TS and CPM; (3) to analyze whether the presence of abnormal VDT as a proxy for DSPN was associated with involvement of pain mechanisms and compare the findings between participants with T1D and T2D.

## Materials and Methods

### Participants

This cross-sectional study includes data originating from 277 participants, of which 56 with T1D and 99 with T2D (DANMARK, ethical approval N-20170045) were recruited from the outpatient clinic at the Department of Endocrinology Aalborg University (Aalborg, Denmark) and through local advertisements. The remaining 122 healthy participants were recruited from three international centers: (I) 99 adults from the University of Pittsburgh Medical Center [Pittsburgh, PA, USA (PRO-17060648)], (II) eight adults from the Johns Hopkins University Medical Center [Baltimore, MD, USA (IRB-00143375)], and (III) 15 adults from Aalborg University Hospital [Aalborg, Denmark (N-20090008)]. Data from healthy participants did not differ depending on the country of origin (see Supplementary Material) and was thus pooled into one database. The QST data from the healthy participants has previously been published by Phillips et al. [19]. All participants provided written informed consent prior to inclusion. Participants with diabetes were eligible for the study if they were >18 years and of European descent. They should have been diagnosed with T1D or T2D for a minimum of 1 year, have verified HbA1C ≥ 6.5%, and receive stable anti-hyperglycemic treatment (insulin or antidiabetics) for at least 1 month prior to inclusion. Exclusion criteria included biochemical abnormalities, symptomatic heart disease or cardiac heart failure, coeliac disease, other neurological or psychiatric diseases, present or previous abuse of alcohol or medicine, use of drugs that affect the nervous system, treatments of endocrinological diseases, and previous chemotherapy. Females who were pregnant or breastfeeding were not allowed to participate. Individuals were not allowed to have participated in other clinical trials 3 months prior to inclusion.

### Quantitative Sensory Testing

QST was performed by centrally trained investigators to evaluate sensory thresholds according to a standardized protocol regarding verbal instructions and technical handling of the QST procedures [19]. A numeric rating scale (NRS) was used to quantify pain: 0 was no pain, and 10 was the worst imaginable pain. All participants underwent the following tests.

#### Phasic Pain

##### Pressure Pain Detection and Tolerance Threshold

A handheld algometer (Algometer type II, Somedic Electronic, Sweden) with a 1 cm^2^ surface area probe was used for muscle pressure. The algometer was applied at the right side of the following skin dermatomes: C5 (below the midline of clavicula), Th10 dorsum, Th10 ventral, L1 (anterior superior iliac crest), and L4 (15 cm above patella on the quadriceps muscle) and pressures were increased continuously by 30 kPa/s. Two thresholds were assessed in kPa: pressure pain detection threshold (pPDT) and pressure pain tolerance threshold (pPTT). For each participant, the sum of all dermatomes was calculated as suggested by Phillips et al. [19]. Similarly, pPDT and pPTT were assessed for the tibial bone using a custom-designed bone probe of 3.1 mm^2^, applied 10 cm distal to patella [27].

### Tonic Pain

#### Immersion of Hand in Cold Water

The cold pressor test was undertaken by immersion of the dominant hand until the wrist in 2°C cold water (MX temperature controller, VWR International, Vienna, Austria or Grant Instruments GD100 Series Stirred Water Baths/Circulators, Cambridge, England or an ice bucket) for a maximum of 120 s. The participants were asked to determine the pain sensation on NRS every 10th second. If the participants experienced intolerable pain and were unable to keep their hand immersed for 120 s, the endurance time was noted (seconds), and a NRS of 10 was carried forward for the remaining period. Analysis was done on the calculated sum of NRS (ΣNRS).

### Central Processing

#### Conditioned Pain Modulation

CPM is a proxy for descending pain modulation [19]. Based on the principle “pain inhibits pain,” CPM investigates how a painful conditioning stimulus (immersion into cold water) modulates a test pain (pPTT on the quadriceps muscle) when the test pain is applied just before and immediately after the conditioning stimulus. To estimate the capacity of the CPM, the relative percentage change between pPTT before and after the cold pressor test was calculated, as described in a previous study [20].

#### Temporal Summation

TS was tested using an 8 mN pinprick stimulator (MRC Systems GmbH, Heidelberg, Germany). The participants were requested to look the other way while a single prick on the midline of the volar side of the right forearm and the abdomen 3–4 cm above the umbilicus was applied. Subsequently, 10 repetitive pinpricks with an inter-stimulus interval of 1 s were carried out at the same anatomical sites guided by a metronome for accurate timing. The participants rated the sensation after the single prick and again after 10 repetitive pricks on NRS. The difference in pain ratings (NRS) between 1 and 10 repetitions was recorded.

#### Vibration Detection Threshold

VDT was measured by a biothesiometer (Bio Medical instruments, Medical Diagnostic Instruments, Newbury, Ohio, USA). While laying down, the biothesiometer was placed perpendicular to the plantar surface of the big toe. Participants were instructed to notify when they first felt the vibration. The test was performed three consecutive times on the left and right big toe, and an average value for each toe was calculated. VDT was only conducted on participants with diabetes and was used as a clinical bedside method as a proxy for estimating the severity of DSPN.

### Statistical Analysis

Normally distributed data are presented as mean ± standard deviation (SD), while non-normally distributed data are presented as median (25th−75th percentile). Binomial data are presented as numbers (percentages). Outliers were excluded based on the 1st and 99th percentile, which was calculated for each parameter. Log-transformation was conducted on the absolute pPDT and pPTT values to obtain a secondary normal distribution. A linear regression model or non-parametric linear regression model with kernel and bootstrapping was used to compare healthy and participants with diabetes and comparison between participants with T1D and T2D. A multivariate regression model was used to adjust for age, BMI, and gender. Co-linearity between age and disease duration existed in participants with T1D (*p* < 0.001) and T2D (*p* = 0.002), and thus data were only adjusted for age and not disease duration. To test whether VDT was associated with the sensory profiling, the participants with T1D and T2D were pooled in one group (diabetes) and used to categorize the cohort into normal VDT (< 18 V), intermediary VDT (18–25 V), or high VDT (>25 V) with comparisons between the groups by use of a Kruskal–Wallis test. Bonferroni correction was conducted to decrease the risk of type-1-error. A significance level of 5% (*p* ≤ 0.05) was adopted and marked with bold in the tables. All analyses were performed using Stata (version 15.1, StataCorp, College Station, Texas, USA).

## Results

### Baseline Characteristics

Baseline characteristics for participants with diabetes and healthy participants are presented in Supplementary Table 1. Participants with diabetes were older [60 years (45–68) vs. 51 years (34–64); *p* < 0.001] and had a higher BMI [29 kg/m^2^ (25–33) vs. 24 kg/m^2^ (23–27); *p* < 0.001] than healthy participants. There was no difference in gender distribution.

### Phasic Pain

#### Pressure Pain Detection Threshold

**Figure 1:**
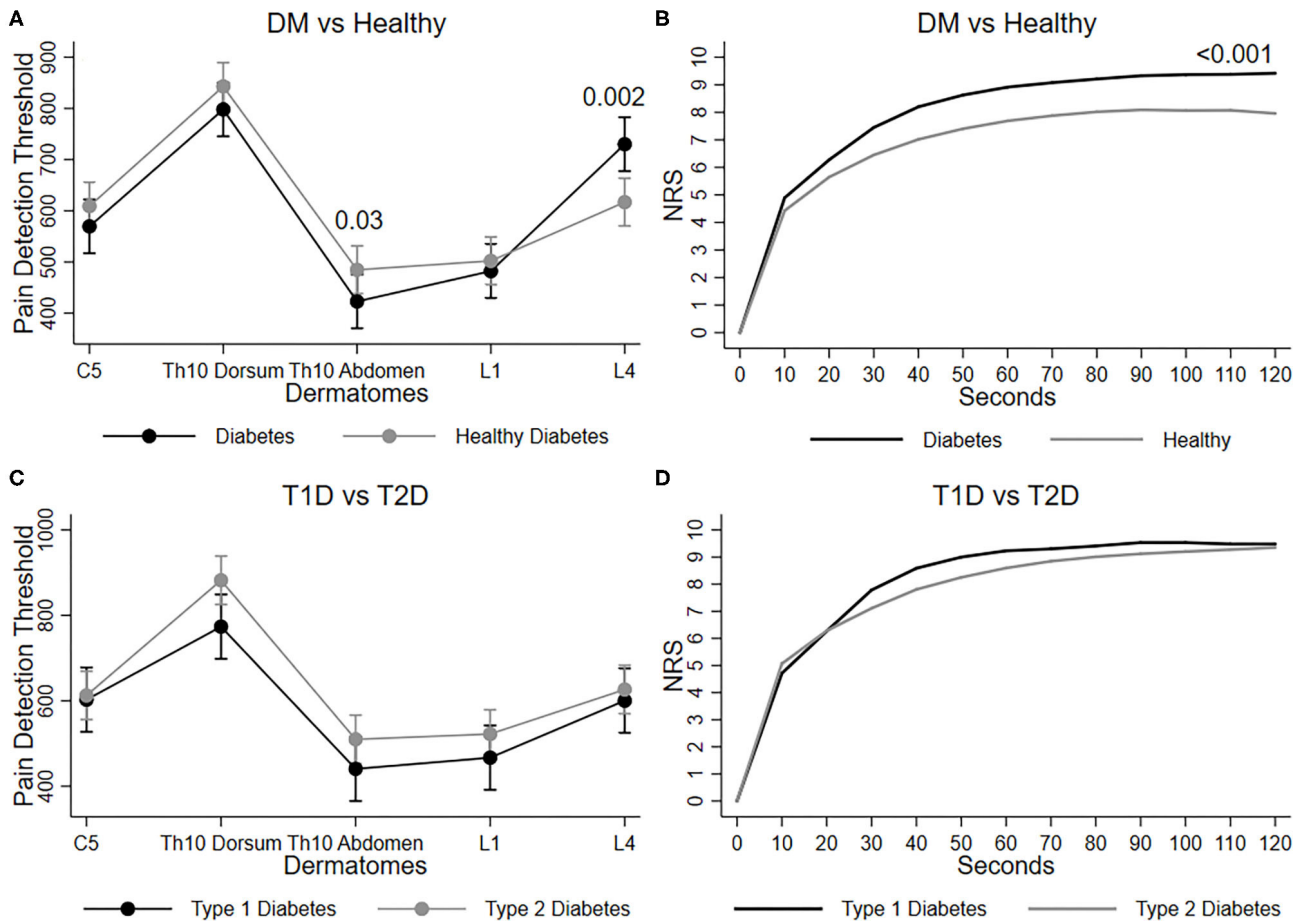
Graphs show mean and 95% confidence intervals of pressure pain tolerance threshold (pPDT) on each dermatome (C5, Th10 dorsum, Th10 Abdomen, L1 and L4) for **(A)** diabetes vs. healthy, and **(C)** Type 1 diabetes vs. type 2 diabetes. Mean NRS score during 120 s immersion of hand in cold water is shown for **(B)** diabetes vs. healthy, and **(D)** type 1 diabetes vs type 2 diabetes.

Participants with diabetes had a higher pPDT at the tibial bone compared to healthy participants (effect size 19 kPa, *p* < 0.01), which was also evident after adjusting (effect size 13 kPa, *p* = 0.01). In the crude analysis, the pPDT sum was not altered in participants with diabetes. However, pPDT at Th10 abdomen was lower (*p* = 0.03), while pPDT at L4 was higher (*p* = 0.002) compared to healthy participants (see Table 1 and Figure 1A).

**Table 1:** Comparison of phasic pain, tonic pain, and central processing between healthy and diabetes.

	**Variables**	**Healthy (*n* = 122)**	**Diabetes (*n* = 155)**	**Unadjusted effect**	***p***	**Adjusted effect**	***p***
Phasic pain	pPDT sum (kPa)	1.613 (1.228–2.154)	1.747 (1.344–2.121)	16 (−154; 186)	0.85	−160 (−319; 0)	0.05
	pPDT L4 Tibia (kPa)	72 (56–96)	98 (76–126)	19 (9, 29)	** <0.01**	13 (3, 24)	**0.01**
	pPTT sum (kPa)	2.748 (2.046–3.686)	2.816 (2.146–3.801)	57 (−244; 358)	0.71	−153 (437; 131)	0.29
	pPTT L4 Tibia(kPa)	118 (86–166)	142 (101–191)	16 (−13; 44)	0.28	4.2 (−25; 33)	0.78
Tonic pain	CP Σpain (ΣNRS)	92 (69–110)	103 (92–112)	13 (8, 18)	** <0.01**	13 (8, 19)	** <0.01**
	CPM capacity (%)	19 (9–30)	18 (1–32)	−1 (−7; 5)	0.73	−1 (−7; 6)	0.83
Central	ΔTS forearm (NRS)^†^	1 (0–2)	0 (0–0)	−0.8 (−1.0; −0.6)	** <0.01**	−0.8 (−1.1; −0.6)	** <0.01**
Processing	ΔTS abdomen (NRS)^†^	1 (0–2)	0 (0–0)	−0.9 (−1.2; −0.7)	** <0.01**	−1.0 (−1.3; −0.7)	** <0.01**

*Comparisons are presented as median (25th−75th percentile). Effects are shown as coefficient (95% confidence intervals). Effects are shown unadjusted and adjusted for age, body mass index, and gender. Significant p-value are marked in bold.*

† *Calculated with a nonparametric regression. pPDT, pain pressure detection threshold; pPTT, pain pressure tolerance threshold; CP, cold pressor; CPM, conditioned pain modulation; TS, temporal summation; NRS, numeric rating scale*.

Participants with T1D had higher pPDT at the tibial bone compared to participants with T2D (effect size 15 kPa, *p* = 0.03), which was even more evident after adjustments (effect size 35 kPa, *p* < 0.01), indicating peripheral hyposensitivity of the tibial bone (Table 2). No difference was found in pPDT for any dermatomes or the pPDT sum (Figure 1C).

**Table 2:** Comparison of phasic pain, tonic pain, and central processing in people with type 1 and 2 diabetes.

**Variable**	**T1D (*n* = 56)**	**T2D (*n* = 99)**	**Unadjusted effect**	***p***	**Adjusted effect**	***p***
pPDT L4 Tibia (kPa)	105 (87–138)	92 (69–120)	15 [2, 28]	**0.03**	35 (17; 53)	** <0.01**
pPTT L4 Tibia (kPa)	177 (120–218)	128 (93–166)	59 (24; 94)	** <0.01**	65 (18; 113)	** <0.01**
CP Σpain (NRS)	104 (94–113)	102 (88–111)	4 (−1; 10)	0.13	6 (−2; 14)	0.12
ΔTS forearm (NRS)^†^	0 (0–1)	0 (0–0)	0.4 (0.1; 0.6)	** <0.01**	0.5 (−0.1; 1.6)	0.26
ΔTS abdomen (NRS)^†^	0 (0–1)	0 (0–0)	0.3 (0.1; 0.6)	**0.02**	0.5 (−0.2; 1.6)	0.33
VDT (V)^†^	12 [10–25]	23 [15–37]	−7 (−12; −3)	** <0.01**	7 (0; 12)	**0.02**

*Comparisons are presented as median (25th−75th percentile). Effects are shown as coefficient (95% confidence intervals). Effects are shown unadjusted and adjusted for age, body mass index, and gender. Significant p-value are marked in bold.*

† *Calculated with a nonparametric regression. T1D, type 1 diabetes mellitus; T2D, type 2 diabetes mellitus; pPDT, pain pressure detection threshold; pPTT, pain pressure tolerance threshold; CP, cold pressor; TS, temporal summation; NRS, numeric rating scale; VDT, vibration detection threshold*.

#### Pressure Pain Tolerance Threshold

No difference between participants with diabetes and healthy was found for the pPTT nor the pPTT sum (see Table 1). Participants with T1D had increased pPTT at the tibial bone compared to participants with T2D (effect size 59 kPa, *p* < 0.01), which also was evident after adjustment (effect size 65 kPa, *p* < 0.01). No difference was found for pPTT for any dermatomes or the pPTT sum (see Table 2).

### Tonic Pain

#### Immersion of Hand in Cold Water

Participants with diabetes experienced increased pain to the cold stimuli during the immersion of the hand in cold water in comparison to healthy participants (effect size 13 ΣNRS, *p* < 0.01), this finding remained after adjustment (effect size 13 ΣNRS, *p* < 0.01; Table 1 and Figures 1B,D).

### Central Processing

#### Conditioned Pain Modulation

There was no difference between participants with diabetes and healthy participants in their capacity to induce CPM.

#### Temporal Summation

Participants with diabetes experienced decreased pain to repetitive pinprick stimuli on the forearm (effect size −0.8 NRS, *p* < 0.01) and the abdomen (effect size −0.9 NRS, *p* < 0.01) compared to healthy participants (see Table 1), which was also evident after adjustments (effect sizes −0.8 NRS and −1.0 NRS, *p* < 0.01).

Those with T1D experienced increased pain to repetitive pinprick on the forearm (effect size 0.4 NRS, *p* < 0.01) and the abdomen (effect size 0.3 NRS, *p* = 0.02) compared to participants with T2D, indicating increased neuronal hyperexcitability. However, these central changes are not evident after adjustment (see Table 2).

#### Vibration Detection Threshold

Participants with T1D had lower VDT compared to participants with T2D (−7 V, *p* < 0.01). After adjustment, VDT was higher in participants with T1D (7 V, *p* = 0.02), indicating more peripheral neuropathy in participants with T1D (see Table 2).

### The Influence of Vibration Detection Threshold

**Figure 2:**
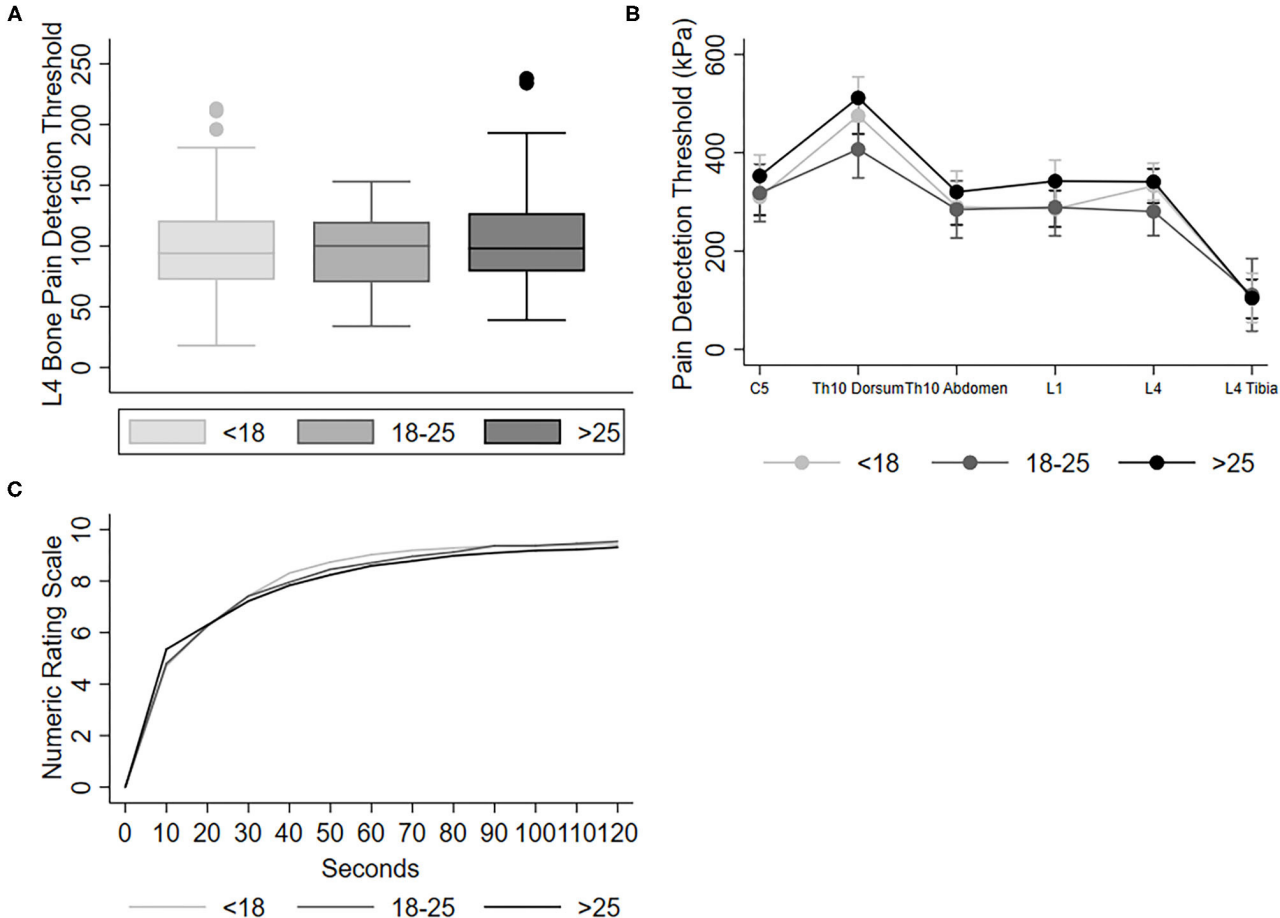
Graphs show **(A)** median pressure pain detection threshold for the tibial bone and **(B)** mean and 95% confidence intervals of pressure pain tolerance threshold on each dermatome (C5, Th10 dorsum, Th10 Abdomen, and L1 and L4) for normal VDT < 18, intermediary VDT 18–25 and high VDT > 25, and **(C)** mean pain score during 120 s immersion of hand in cold water. All pressure was obtained with a 1 cm^2^ probe on muscle tissue, except L4 Tibia which were obtained with a 3.1 mm^2^ probe on the bone, accounting for the lower pressure at this point.

In the three categories of VDT (normal, intermediary, and high VDT), no difference was seen in pPDT or pPTT at the tibial bone (Figure 2A) or in the remaining dermatomes (Figure 2B). Also, there were no differences between the perceived pain categories during tonic pain (Figure 2C), TS, or CPM capacity. Details are shown in Table 3.

**Table 3:** Comparison of phasic pain, tonic pain, and central processing in diabetes based on vibration detection threshold.

	**Variables**	**Normal VDT (*n* = 72)**	**Intermediary VDT (*n* = 29)**	**High VDT (*n* = 54)**	***p***
Phasic pain	pPDT L4 Tibia (kPa)	94 (72–121)	100 (70–120)	98 (79–127)	0.86
	pPTT L4 Tibia (kPa)	157 (112–214)	124 (96–176)	134 (97–166)	0.06
Tonic pain	CP Σpain (NRS)	103 (93–111)	103 (88–113)	104 (85–114)	0.96
Central processing	ΔTS forearm (NRS)	0 (0–0)	0 (0–0)	0 (0–0)	0.31
	ΔTS abdomen (NRS)	0 (0–0)	0 (0–0)	0 (0–0)	0.12

*Data is presented as median (IQR). VDT, vibration detection threshold (normal: < 18, intermediary: 18–25, high: >25), NRS, numeric rating scale, pPDT, pain pressure detection threshold; pPTT, pain pressure tolerance threshold; CP, cold pressor; TS, temporal summation*.

## Discussion

This study characterizes perceived phasic and tonic pain, CPM, and TS in T1D, T2D, and healthy participants. In the comparison between participants with diabetes and healthy, we showed paradoxical co-existence of hypoesthesia to phasic bone pressure, decreased TS, and increased pain to cold stimuli, also after adjustments for age, BMI, and gender. In comparison to T2D, participants with T1D were hypoalgesic to bone pain and had lower VDT. When categorized based on VDT (normal, intermediary, and high), a rough proxy for Aβ-fiber function, no associations were shown with experienced sensory responses to phasic or tonic pain, challenging the hypothesis of an association between VDT and severity of DSPN. The use of this relatively simple QST protocol revealed the involvement of peripheral and central pain mechanisms and emphasized differences in T1D and T2D, adding information to the current clinical need for thorough phenotyping and identification of future clinical endpoints.

### Phasic Pain

The periosteum of the tibia is richly innervated by Aδ- and C-fibers and is known to be sensitive to noxious pressure stimulations. Tibia is relatively exposed and is ideal for applying and reliably assessing bone pain, with a negligible skin component [27]. Even though no previous studies have investigated bone pain in diabetes, we expected and confirmed hypoesthesia due to DSPN rather than hyperalgesia to nociceptive stimuli as seen in other neuropathic conditions. No differences in sensory responses to pressure representing different dermatomes were shown. Hence no differences in pPDT on the quadriceps muscle were shown, which is in accordance with other studies using the same handheld algometer [9, 25]. This negative finding may emphasize that distal axonopathy (glove and stocking distribution) is more pronounced than neuro-inflammation or central alterations in the corresponding L4 dermatome. Participants with T1D had increased tibial pPDT and pPTT compared to T2D (also after adjustment), indicating larger deafferentation of the periosteum than in T2D.

### Tonic Pain

Participants with diabetes experienced increased pain during immersion of the hand in cold water in comparison to healthy. The influence of gender is supported by a meta-analysis performed by Fillingim et al. [28], justifying the adjustment. Further, it could be speculated that changes in the peripheral sensory nerve fibers due to the presence of DSPN represent the paradox of decreased peripheral nerve function but increased pain sensation. Alternatively, it could be due to decreased peripheral blood circulation because of impaired vasomotor function [29]. As such, vasomotor impairment could lead to a lack of protective thermoregulation, consequently increasing the experience of cold pain. Finally, especially T1D is often accompanied by the daily tasks of multiple blood glucose measurements through finger-prick. Over the years, this may damage the sensation in the fingertips; however, no differences between T1D and T2D were seen.

### Central Processing

Dynamic QST is cheap and easily applicable, and thus it has been widely used to investigate central pain mechanisms where widespread hyperalgesia is a well-known phenomenon, e.g., in chronic pancreatitis [20, 30, 31]. In contrast, limited and contradictory data regarding the involvement of such central mechanisms in diabetes exist. CPM efficiency in painful diabetic neuropathy patients was used to predict the efficacy of adequate response to duloxetine, whereas other dynamic pain modulatory parameters such as TS could not predict drug response [32]. Furthermore, longer diabetes duration has been shown to associate with efficient CPM response and diminished TS [17]. In contrast to these, a relatively small study reported no differences in TS between diabetes and healthy subjects [25], while our study showed decreased TS. The different findings may reflect deafferentation of the distal sensory nerves, indicating an abnormal response to pin-prick *per se*. If this is the case, the TS responses may have been hampered by the use of different methodologies, e.g., the use of von Frey filaments in contrast to pinprick on the forearm and abdomen used in the current study. The crude analysis indicated a difference in TS between T1D and T2D. However, the pinprick responses from the abdomen were affected by BMI as the effect was abolished after adjustments. This is in accordance with previous studies showing that obesity influences pain sensitivity, especially in areas with increased subcutaneous adipose, such as the abdomen [33, 34]. When conducted appropriately, TS is a central phenomenon in which repeated stimuli at an equal-intensity and specific frequency produce an increased experienced pain in patients with neural hyperexcitability. It has been suggested that sub-threshold impulses from two or more central synapses trigger the action potential because of synergistic interactions. If this is correct, previous data on prolonged synaptic and central neuronal conduction time in people with diabetes may support that TS is diminished [35].

### Vibration Detection Threshold

VDT is a well-established clinical method to screen for sensory abnormalities and impairment of the large Aβ-fibers in DSPN [36]. Even though participants with T2D at first glance seemed to have a higher VDT than participants with T1D, indicating more pronounced peripheral neuronal damage [37], this trend was inverted after adjustment for age, BMI, and gender, emphasizing an age phenomenon to the test: axons typically have been exposed to fluctuating glycemic levels over a longer period in T1D. This outweighs the fact that neuronal damage in T2D may already be seen at the time of diagnosis because neuropathy represents the progression of systemic capillary dysfunction, and disease duration is therefore reported falsely low [29].

### Influence of Vibration Detection Thresholds

VDT is widely used to screen for the severity of DSPN; however, the threshold is independent of the participant's phenotype, e.g., with/without the presence of painful neuropathy [38]. To the best of our knowledge, no existing studies have used the VDT to categorize data into normal, intermediary, and high VDT corresponding to the severity of progressive neuronal damage to explore whether these would predict involvement of differentiated pain mechanisms. To our surprise, we found no associations between VDT and involvement of the pain mechanisms. It would have seemed plausible that high VDT was associated with hyposensitivity, but the finding could possibly reflect that deafferentation and impairment of Aβ-fibers (vibration) and Aδ-fibers (mechanical pressure) does not run in parallel. Notably, the QST assessments have provided a more complex and differentiated profile of the involved fibers unraveling the co-existence of hypoesthesia and central hyperexcitability. Thus further exploration of these mechanistic pathways may lead to an improved understanding of the paradoxical clinical phenotypes.

### Limitations

This study is not without limitations. Firstly, the cohort consisted of participants investigated at different sites, and though testing protocols were centrally standardized, this risk of inter-rater differences increases. Moreover, pain is a global feature of the human experience and data originating from different continents may be influenced by cultural, societal, and educational rules. However, no differences in the healthy data were found between countries (see Supplementary Material). Additionally, the diabetes cohort investigated in Denmark consisted of participants of northern European descent, in contrast to the healthy cohort originating from the US, where racial diversity is more common [39, 40]. Secondly, the majority of adults with diabetes have complications and co-morbidities with several appointments in the healthcare system [2, 41]. Therefore, selection bias cannot be ruled out, favoring participants with more resources and lesser affection by DSPN than the generalized diabetes population. If so, it will only strengthen our discrepancies between diabetes and healthy, supported by previous studies. We found it optimal to use the standardized QST-protocol that we are very familiar with, but it should be noted that it is primarily developed to determine the involvement of central hyperalgesia in patients with chronic painful pancreatitis [19]. Future protocols should ideally assess pro- and anti-inflammatory cytokines in order to determine the role of neuroinflammation in diabetes. Despite these limitations, the protocol has revealed clinically meaningful differentiated characteristics between T1D and T2D and healthy, revealing a complex characterization of the pain system in diabetes.

## Conclusion

This study confirmed the usefulness of a standardized QST-protocol applied in diabetes to fully appreciate and recognize the paradoxical co-existence of hypo- and hyperalgesia. The involvement of different pain mechanisms in diabetes showed paradoxical co-existence of hypoesthesia to phasic bone pressure and hyperalgesia to tonic cold pain when compared to healthy, even after adjustments. Furthermore, the protocol revealed differences in responses to bone pain between T1D and T2D, which may be useful in identifying future clinical endpoints, possibly supported by a mechanistic approach to fully elucidate the involvement of Aβ-fibers, Aδ-fibers, and C-fibers. The accuracy of the vibration detection threshold is challenged as a valid measure of severity of distal symmetrical polyneuropathy and emphasizes that QST may provide the needed information to phenotype patients and characterize the complex alterations of the pain system in the clinic.

## Data Availability Statement

The data that support the findings of this study are available from the corresponding author upon reasonable request.

## Ethics Statement

The studies involving human participants were reviewed and approved by North Denmark Region Committee on Health Research Ethics (N-20170045). The patients/participants provided their written informed consent to participate in this study.

## Author Contributions

CB and AD: study design and original idea. A-MW, SO, AP, and MF: collected data. LH, CF, SO, and A-MW: analyzed the data. LH and CF: wrote the first draft. CB: guarantor of the work, with full access to all the data in the study and takes responsibility for the data integrity and data analysis accuracy. All authors interpreted the data, contributed to the final manuscript, and critically reviewed the manuscript.

## Conflict of Interest

The authors declare that the research was conducted in the absence of any commercial or financial relationships that could be construed as a potential conflict of interest.

## References

[1] Peltier A Goutman SA Callaghan BC. Painful diabetic neuropathy. BMJ. (2014) 348:g1799. 10.1136/bmj.g1799 24803311

[2] Zaccardi F Webb DR Yates T Davies MJ. Pathophysiology of type 1 and type 2 diabetes mellitus: a 90-year perspective. Postgrad Med J. (2016) 92:63–9. 10.1136/postgradmedj-2015-133281 26621825

[3] Feldman EL Callaghan BC Pop-Busui R Zochodne DW Wright DE Bennett DL. Diabetic neuropathy. Nat Rev Dis Prim. (2019) 5:41. 10.1038/s41572-019-0092-1 31197153

[4] Fowler MJ. Microvascular and macrovascular complications of diabetes. Clin Diabetes. (2008) 26:77–82. 10.2337/diaclin.26.2.77

[5] Little AA Edwards LJ Feldman LE. Diabetic neuropathies. Neurologist. (2005) 11:63–79. 10.1097/01.nrl.0000156314.24508.ed 15733329

[6] Brock C Hansen CS Karmisholt J Møller HJ Juhl A Farmer AD. Liraglutide treatment reduced interleukin-6 in adults with type 1 diabetes but did not improve established autonomic or polyneuropathy. Br J Clin Pharmacol. (2019) 85:2512–23. 10.1111/bcp.14063 31338868 PMC6848951

[7] Tesfaye S Selvarajah D Gandhi R Greig M Shillo P Fang F. Diabetic peripheral neuropathy may not be as its name suggests: evidence from magnetic resonance imaging. Pain. (2016) 157(Suppl. 1):S72–S80. 10.1097/j.pain.0000000000000465 26785159

[8] Gylfadottir SS Christensen DH Nicolaisen SK Andersen H Callaghan BC Itani M. Diabetic polyneuropathy and pain, prevalence, and patient characteristics: a cross-sectional questionnaire study of 5,514 patients with recently diagnosed type 2 diabetes. Pain. (2020) 161:574–83. 10.1097/j.pain.0000000000001744 31693539 PMC7017941

[9] Brock C Søfteland E Frøkjær JB Drewes AM Arendt-Nielsen L. Associations between sensorimotor, autonomic and central neuropathies in diabetes mellitus. J Diabetes Metab. (2014) 05:1–6. 10.4172/2155-6156.1000390

[10] Pop-Busui R Ang L Holmes C Gallagher K Feldman EL. Inflammation as a therapeutic target for diabetic neuropathies. Curr Diab Rep. (2016) 16:1–10. 10.1007/s11892-016-0727-5 26897744 PMC5127166

[11] Walk D Sehgal N Moeller-Bertram T Edwards RR Wasan A Wallace M. Quantitative sensory testing and mapping a review of nonautomated quantitative methods for examination of the patient with neuropathic pain. Clin J Pain. (2009) 25:632–40. 10.1097/AJP.0b013e3181a68c64 19692806

[12] Themistocleous AC Ramirez JD Shillo PR Lees JG Selvarajah D Orengo C. The pain in Neuropathy Study (PiNS): a cross-sectional observational study determining the somatosensory phenotype of painful and painless diabetic neuropathy. Pain. (2016) 157:1132–45. 10.1097/j.pain.0000000000000491 27088890 PMC4834814

[13] Raputova J Srotova I Vlckova E Sommer C Uceyler N Birklein F. Sensory phenotype and risk factors for painful diabetic neuropathy: a cross-sectional observational study. Pain. (2017) 158:2340–53. 10.1097/j.pain.0000000000001034 28858986 PMC5690294

[14] Sierra-Silvestre E Somerville M Bisset L Coppieters MW. Altered pain processing in patients with type 1 and 2 diabetes: systematic review and meta-analysis of pain detection thresholds and pain modulation mechanisms. BMJ Open Diabetes Res Care. (2020) 8:1–9. 10.1136/bmjdrc-2020-001566 32868312 PMC7462232

[15] Vollert J Maier C Attal N Bennett DLH Bouhassira D Enax-Krumova EK. Stratifying patients with peripheral neuropathic pain based on sensory profiles: algorithm and sample size recommendations. Pain. (2017) 158:1446–55. 10.1097/j.pain.0000000000000935 28595241 PMC5515640

[16] Maier C Baron R Tölle TR Binder A Birbaumer N Birklein F. Quantitative sensory testing in the German Research Network on Neuropathic Pain (DFNS): somatosensory abnormalities in 1236 patients with different neuropathic pain syndromes. Pain. (2010) 150:439–50. 10.1016/j.pain.2010.05.002 20627413

[17] Phillips TJC Brown M Ramirez JD Perkins J Woldeamanuel YW De Williams ACC. Sensory, psychological, and metabolic dysfunction in HIV-associated peripheral neuropathy: a cross-sectional deep profiling study. Pain. (2014) 155:1846–60. 10.1016/j.pain.2014.06.014 24973717 PMC4165602

[18] Üçeyler N Vollert J Broll B Riediger N Langjahr M Saffer N. Sensory profiles and skin innervation of patients with painful and painless neuropathies. Pain. (2018) 159:1867–76. 10.1097/j.pain.0000000000001287 29863528

[19] Phillips AE Faghih M Kuhlmann L Larsen IM Drewes AM Singh VK. A clinically feasible method for the assessment and characterization of pain in patients with chronic pancreatitis. Pancreatology. (2019) 20:25–34. 10.1016/j.pan.2019.11.007 31787527

[20] Kuhlmann L Olesen SS Olesen AE Arendt-Nielsen L Drewes AM. Mechanism-based pain management in chronic pancreatitis-is it time for a paradigm shift? Expert Rev Clin Pharmacol. (2019) 12:249–58. 10.1080/17512433.2019.1571409 30664364

[21] Arendt-Nielsen L Morlion B Perrot S Dahan A Dickenson A Kress HG. Assessment and manifestation of central sensitisation across different chronic pain conditions. Eur J Pain. (2018) 22:216–41. 10.1002/ejp.1140 29105941

[22] Mackey IG Dixon EA Johnson K Kong JT. Dynamic quantitative sensory testing to characterize central pain processing. J Vis Exp. (2017) 2017:1–9. 10.3791/54452 28287532 PMC5407598

[23] Olesen SS Brock C Krarup AL Funch-Jensen P Arendt-Nielsen L Wilder-Smith OH. Descending inhibitory pain modulation is impaired in patients with chronic pancreatitis. Clin Gastroenterol Hepatol. (2010) 8:724–30. 10.1016/j.cgh.2010.03.005 20304100

[24] Niesters M Proto PL Aarts L Sarton EY Drewes AM Dahan A. Tapentadol potentiates descending pain inhibition in chronic pain patients with diabetic polyneuropathy. Br J Anaesth. (2014) 113:148–56. 10.1093/bja/aeu056 24713310

[25] Søfteland E Brock C Frøkjær JB Brøgger J Madácsy L Gilja OH. Association between visceral, cardiac and sensorimotor polyneuropathies in diabetes mellitus. J Diabetes Complications. (2014) 28:370–7. 10.1016/j.jdiacomp.2013.10.009 24355661

[26] Granovsky Y Nahman-Averbuch H Khamaisi M Granot M. Efficient conditioned pain modulation despite pain persistence in painful diabetic neuropathy. Pain Rep. (2017) 2:1–7. 10.1097/PR9.0000000000000592 29392208 PMC5741298

[27] Andresen T Pfeiffer-Jensen M Brock C Drewes AM Arendt-Nielsen L. A human experimental bone pain model. Basic Clin Pharmacol Toxicol. (2013) 112:116–23. 10.1111/bcpt.12002 22925354

[28] Fillingim RB King CD Ribeiro-Dasilva MC Rahim-Williams B Riley JL. Sex, gender, and pain: a review of recent clinical and experimental findings. J Pain. (2009) 10:447–85. 10.1016/j.jpain.2008.12.001 19411059 PMC2677686

[29] Østergaard L Finnerup NB Terkelsen AJ Olesen RA Drasbek KR Knudsen L. The effects of capillary dysfunction on oxygen and glucose extraction in diabetic neuropathy. Diabetologia. (2015) 58:666–77. 10.1007/s00125-014-3461-z 25512003 PMC4351434

[30] Kramer HH Rolke R Bickel A Birklein F. Thermal thresholds predict painfulness of diabetic neuropathies. Diabetes Care. (2004) 27:2386–91. 10.2337/diacare.27.10.2386 15451905

[31] Nuwailati R Curatolo M LeResche L Ramsay DS Spiekerman C Drangsholt M. Reliability of the conditioned pain modulation paradigm across three anatomical sites. Scand J Pain. (2019) 20:283–96. 10.1515/sjpain-2019-0080 31812949

[32] Yarnitsky D Granot M Nahman-Averbuch H Khamaisi M Granovsky Y. Conditioned pain modulation predicts duloxetine efficacy in painful diabetic neuropathy. Pain. (2012) 153:1193–8. 10.1016/j.pain.2012.02.021 22480803

[33] Tashani OA Astita R Sharp D Johnson MI. Body mass index and distribution of body fat can influence sensory detection and pain sensitivity. Eur J Pain. (2017) 21:1186–96. 10.1002/ejp.1019 28263427

[34] Price RC Asenjo JF Christou NV Backman SB Schweinhardt P. The role of excess subcutaneous fat in pain and sensory sensitivity in obesity. Eur J Pain. (2013) 17:1316–26. 10.1002/j.1532-2149.2013.00315.x 23576531

[35] Nissen TD Meldgaard T Nedergaard RW Juhl AH Jakobsen PE Karmisholt J. Peripheral, synaptic and central neuronal transmission is affected in type 1 diabetes. J Diabetes Complications. (2020) 34:107614. 10.1016/j.jdiacomp.2020.107614 32571684

[36] Veves A Backonja M Malik RA. Painful diabetic neuropathy: epidemiology, natural history, early diagnosis, and treatment options. Pain Med. (2008) 9:660–74. 10.1111/j.1526-4637.2007.00347.x 18828198

[37] Sierra-Silvestre E Bisset L Coppieters MW. Altered pain processing in people with type I and II diabetes: a protocol for a systematic review and meta-analysis of pain threshold and pain modulation mechanisms. Syst Rev. (2018) 7:222. 10.1186/s13643-018-0895-2 30518431 PMC6280339

[38] Tesfaye S Boulton AJM Dyck PJ Freeman R Horowitz M Kempler P. Diabetic neuropathies: update on definitions, diagnostic criteria, estimation of severity, and treatments. Diabetes Care. (2010) 33:2285–93. 10.2337/dc10-1303 20876709 PMC2945176

[39] Kim HJ Greenspan JD Ohrbach R Fillingim RB Maixner W Renn CL. Racial/ethnic differences in experimental pain sensitivity and associated factors - Cardiovascular responsiveness and psychological status. PLoS One. (2019) 14:e0215534. 10.1371/journal.pone.0215534 30998733 PMC6472780

[40] Kim HJ Yang GS Greenspan JD Downton KD Griffith KA Renn CL. Racial and ethnic differences in experimental pain sensitivity. Pain. (2017) 158:194–211. 10.1097/j.pain.0000000000000731 27682208

[41] Olesen AE Farmer AD Olesen SS Aziz Q Drewes AM. Management of chronic visceral pain. Pain Manag. (2016) 6:469–86. 10.2217/pmt-2015-0011 27256577

