# Supplementary material for: Simple Quantitative Sensory Testing Reveals Paradoxical Co-existence of Hypoesthesia and Hyperalgesia in Diabetes
Source: Front Pain Res (Lausanne). 2021 Jun 28;2:701172. doi: 10.3389/fpain.2021.701172 (PMC8915693; doi:10.3389/fpain.2021.701172)
Supplement: Supplementary file 1 [file Table_1.DOCX]

Supplementary Material

| Variables | Diabetes  (n = 155) | Healthy  (n = 122) | p |  |
| --- | --- | --- | --- | --- |
| Gender (male) | 88 (57) | 61 (50) | 0.26 |  |
| Age (years) | 60 (45-68) | 51 (34-64) | <0.001 | * |
| Body Mass Index (kg/m^2^) | 29 (25-33) | 24 (23-27) | <0.001 | * |
| Years from diagnosis (years) | 15 (7-21) | N/A |  |  |
| Right-handed (yes) | 141 (91) | 110 (90) | 0.82 |  |
| Nicotine use (yes) | 62 (40) | N/A |  |  |
| Hemoglobin A1c (mmol/mol) | 57 (49-64) | N/A |  |  |
| Systolic blood pressure (mmHg) | 136 ± 16 | N/A |  |  |
| Diastolic blood pressure (mmHg) | 75 ± 9 | N/A |  |  |
| Heartbeat (beats/min) | 70 ± 9 | N/A |  |  |
| Use of long acting insulin (yes) | 59 (38) | N/A |  |  |
| Long acting insulin dose (ie/day) | 29 (20-45) | N/A |  |  |
| Use of short acting insulin (yes) | 43 (28) | N/A |  |  |
| Short acting insulin dose (ie/day) | 22 (14-30) | N/A |  |  |
| Use of insulin pump (yes) | 21 (14) | N/A |  |  |
| Insulin pump basal dose (ie/day) | 22 ± 5 | N/A |  |  |
| Insulin pump bolus dose (ie/day) | 22 ± 8 | N/A |  |  |
| Use of Metformin (yes) | 81 (52) | N/A |  |  |
| Data is presented as mean ± SD or median (IQR) based on normality distribution and as number (%) for binorminal data. Nicotine use is defined as present or previously smoking.  T1D: type 1 diabetes mellitus, T2D: type 2 diabetes mellitus, N/A: non-applicable | | | | |

Table 1 – Baseline Characteristics
